# Severe obesity and fitness in New York City public school youth, 2010–2018

**DOI:** 10.1186/s12889-023-15267-w

**Published:** 2023-02-16

**Authors:** Cody D. Neshteruk, Sophia E. Day, Kevin J. Konty, Sarah C. Armstrong, Asheley C. Skinner, Emily M. D’Agostino

**Affiliations:** 1 Department of Population Health Sciences, Duke University School of Medicine, 215 Morris Street, Suite 210, Durham, NC 27701 USA; 2 New York City Department of Health and Mental Hygiene, Office of School Health, New York, NY USA; 3 Duke Clinical Research Institute, Duke University, Durham, North Carolina USA; 4 Department of Pediatrics, Duke University School of Medicine, Durham, North Carolina USA; 5 Department of Orthopaedic Surgery, Duke University School of Medicine, Durham, North Carolina USA

**Keywords:** Obesity, Children, Pediatrics, Physical activity, Cardiovascular health, Exercise, Fitness, Fitnessgram

## Abstract

**Background:**

Obesity is associated with poorer youth fitness. However, little research has examined the magnitude of this relationship in youth with severe obesity. Therefore, we sought to determine the relationship between increasing weight status and fitness within a sample of children and adolescents from New York City public schools.

**Methods:**

This study utilized longitudinal data from the NYC Fitnessgram dataset years 2010–2018. Height and weight along with fitness were measured annually during physical education classes. Severity of obesity was defined using body mass index relative to the 95th percentile and then categorized into classes. A composite measure of fitness was calculated based on scores for three fitness tests: aerobic capacity, muscular strength, and muscular endurance. To examine the weight status-fitness relationship, repeated measures mixed models with random-intercepts were constructed. Stratified models examined differences by demographic factors.

**Results:**

The sample included 917,554 youth (51.8% male, 39.3% Hispanic, 29.9% non-Hispanic Black, 14.0%, 4.6%, and 1.6% class I, II and III obesity, respectively). Compared to youth with healthy weight, increasing severity of obesity was associated with decreased fitness: overweight (β = − 0.28, 95% CI:-0.29;-0.28), class I obesity (β = − 0.60, 95% CI:-0.60; − 0.60), class II obesity (β = − 0.94, 95% CI:-0.94; − 0.93), and class III obesity (β = − 1.28; 95% CI:-1.28; − 1.27). Stratified models showed the association was stronger among male and non-Hispanic White youth.

**Conclusion:**

Findings revealed that more severe obesity was associated with lower fitness. Future research is needed to develop targeted interventions to improve fitness in youth with obesity.

## Introduction

Physical activity (PA) is necessary for children’s healthy growth and development, yet worldwide, few children and adolescents obtain sufficient PA [1]. In the United States (US), less than half of children and even fewer adolescents meet the national PA recommendation of at least 60 minutes of moderate-to-vigorous PA per day [2]. Low levels of PA in youth correspond to low physical fitness, which is a strong, independent predictor of both current and future health [3, 4]. During childhood and adolescence, higher fitness is associated with reduced cardiovascular disease risk, better mental health, and improved academic performance [5]. However, temporal trends in youth fitness show that fitness has declined among youth worldwide [6]. For instance, only 42% of US children 12–15 years obtain optimal cardiorespiratory fitness levels in 2012, a decline from 52% in 1999 [7]. Furthermore, evidence shows that there are wide disparities in youth fitness across demographic characteristics including sex, age, race/ethnicity and income [6, 8–14]. Youth with obesity also experience disparities in fitness, often having lower fitness compared to their healthy weight peers [7, 15].

To date, the majority of studies that have examined the relationship between weight status and fitness use a single category of obesity, rather than considering the severity of obesity, largely in part due to small samples of youth with severe obesity [7, 15–17]. Examining the relationship between obesity and fitness across different classes of obesity is especially relevant given the rise in the prevalence of severe obesity and the associated cardiovascular and metabolic risks experienced by youth with severe obesity [18–20]. Furthermore, few studies have explored the weight status-fitness relationship across demographic factors. As such, key questions remain regarding the relationship between increasing severity of obesity and fitness, and how this relationship is modified by demographic factors. To answers these questions, we used a large, diverse dataset of New York City (NYC) public school youth captured over 8 years. Previous research has documented the prevalence of obesity and poor fitness in these youth [14, 21]. Therefore, the purpose of this study was to 1) test the longitudinal association between weight status and fitness and 2) examine the moderating effect of demographic characteristics on this relationship.

## Methods

### Data source

Data for this study were drawn from the NYC Fitnessgram dataset jointly managed by the NYC Department of Education (DOE) and Department of Health and Mental Hygiene (DOHMH). A detailed description of the dataset and methods used in the NYC Fitnessgram are available elsewhere [22]. Briefly, the NYC Fitnessgram dataset includes all NYC students enrolled in a general education (i.e., non-charter or special education) public school beginning in 2006–07. Height and weight measures are available for all students in kindergarten-grade 12, and fitness measures are available for students in grades 4–12. Student measurements are collected by teachers during physical education classes using a standard protocol based on the Cooper Institute’s Fitnessgram, a reliable and valid assessment that relates to present and future child health outcomes [23]. Child-level student demographic data were drawn from NYC DOE student enrollment records linked to Fitnessgram data by a unique identifier. All protocols were approved by the DOHMH Institutional Review Board, which determined this study was exempt from obtaining written informed consent.

### Participants

Students enrolled in grades 4–12 during the 2010–11 through 2017–18 school years (*n* = 1,393,987) were eligible for this study due to a higher percentage of complete data during this time period. To be included, youth had to have at least 1 year with both height and weight measurements and scores for three fitness assessments (i.e. Progressive Aerobic Cardiovascular Endurance Run (PACER), curl-ups, and push-ups) in the following year (i.e., lagging fitness scores to weight status). For each record, a child must also have had a non-missing date of birth and sex, and age at end of the first school year between 9 and 19 years.

### Exposure

The primary exposure was weight status defined by age- and sex-specific body mass index (BMI) percentiles based on height and weight collected annually, and in accordance with the Centers for Disease Control and Prevention (CDC) growth charts [24, 25]. Age in months was calculated from the measurement date and students’ date of birth was drawn from school enrollment records. Extreme or biologically implausible values were identified for height, weight, weight-for-height, and BMI using CDC’s age- and sex-specific criteria [25]. An observation identified as biologically implausible for a student in a single school year was excluded only for that year. Weight status was defined as follows: underweight (BMI <5th percentile), healthy weight (5th ≤ BMI < 85th percentile), overweight (85th ≤ BMI < 95th percentile), and obese (BMI ≥ 95th percentile). Obesity was stratified in accordance with previous reports [25, 26]. Specifically, class I obesity was defined as BMI ≥95th percentile or a BMI ≥30; class II obesity was defined as a BMI ≥120% of the 95th percentile or a BMI of ≥35; and class III obesity was defined as a BMI ≥140% of the 95th percentile or a BMI of ≥40 or greater.

### Outcome

The outcome was age- and sex-specific fitness composite z-scores based on the sum of scores for the PACER (assessing aerobic capacity) and curl-up and push-up (assessing muscular strength and endurance) tests [22, 23]. The NYC Fitnessgram includes a subset of tests chosen from the available battery of Fitnessgram tests including the PACER, push-up, curl-up, sit and reach and trunk lift test. For the PACER, push-up and curl-up tests, scores are converted to z-scores to account for expected improvements in performance with increasing age and sex and are subsequently used to create a composite fitness z-score. All z-scores are created by pooling youth actively enrolled in the NYC DOE during the study period, and pooling PACER, curl-up, and push-up valid scores and accounting for the sex*age in months. Only valid PACER, curl-up, and push-up scores were included for each year of data from each child. The three fitness z-scores are then summed for all records with all three valid scores and transformed to z-scores using the standard normal equation to yield a total fitness z-score. The component scores were also examined individually and findings are presented elsewhere [27].

### Covariates

Youth’s sex, grade, race/ethnicity, eligibility for free/reduced price lunches, country of birth, and English language learner status were drawn from NYC DOE school enrollment records. Grade was categorized into 4-8th grade (late elementary and middle school) or 9-12th grade (high school). Race/ethnicity was grouped into five categories: non-Hispanic White, non-Hispanic Black, Hispanic, Asian/Pacific Islander, and other (including multiple races, Native American, and refusal to provide). To categorize youth in terms of socioeconomic status, individual child household poverty (high vs. low) was based on baseline child eligibility/non-eligibility for free/reduced-price school meals through the National School Lunch Program, which provides meal assistance according to household income at or below 185% of the federal poverty level [28]. Country of birth was categorized into foreign-born or US-born. English language learner status was classified as yes/no.

### Statistical analyses

Descriptive statistics were computed to summarize sample characteristics. Means and standard deviations were calculated for fitness composite z-scores by weight status across sample characteristics (sex, race/ethnicity, grade level, poverty status, country of birth, and English language learner status). Next, repeated measures mixed models with random intercepts were fit to the data to test the association between weight class and fitness. To ensure temporality between the exposure (weight class) and outcome (fitness), we used one-year lagged fitness z-scores as the outcome. To account for clustering, annual observations were clustered at the census tract level. To further understand the weight status-fitness relationship in demographic subgroups, stratified models were constructed for each sample characteristic listed above. All adjusted models included sex, grade level, race/ethnicity, poverty status, country of birth, English language learner status and time (continuous variable) as covariates. Statistical analyses were performed using SAS v.9.4.

## Results

The final analytic sample included 917,554 youth contributing 3,586,585 observations from the 2010–11 through 2017–18 school years, with individual youth having one to eight repeated annual observations and 58% of the sample having at least five repeated annual observations. Table 1 shows the demographic characteristics of the sample. Slightly more than half of youth were male (51.8%) and most were Hispanic (39.3%) and non-Hispanic Black (29.9%), eligible for free/reduced price meals (70.5%) and born outside of the US (72.1%). The majority of observations were obtained during elementary and middle school (58.7%) and the median age was 15.5 years. During the study period, 18.6%, 14.0%, 4.6%, and 1.6% of youth had overweight, class 1, class 2, and class 3 obesity respectively.

**Table 1:** Sample characteristics for NYC Public School Children 2010–2018 (*n* = 917,554)

Characteristic	n (%) ^a^
Sex	
Male	475,441 (51.8)
Female	442,112 (48.2)
Race/ethnicity	
Non-Hispanic White	123,426 (13.5)
Non-Hispanic Black	274,115 (29.9)
Hispanic	360,498 (39.3)
Asian/Pacific Islander	146,353 (16.0)
Other ^b^	13,162 (1.4)
Free/reduced meal status	
Yes	646,687 (70.5)
No	270,867 (29.5)
Country of birth	
Foreign born	661,140 (72.1)
US born	256,414 (28.0)
English language learner	
Yes	128,671 (14.0)
No	788,883 (86.0)
Grades (all years) ^c^	
4th – 8th	2,105,597 (58.7)
9th – 12th	1,480,988 (41.3)
Weight status (all years) ^c^	
Underweight	126,803 (3.5)
Healthy weight	2,070,361 (57.7)
Overweight	665,694 (18.6)
Class I obesity	501,114 (14.0)
Class II obesity	165,715 (4.6)
Class III obesity	56,898 (1.6)

*Abbreviations*: *NYC* New York City

^a^Students contributed 3,586,585 observations from 2010 to 2018

^b^Other includes mixed race, Native American, and refusal to provide

^c^Because there were multiple observations at different time points, these categories are presented for all observations

Table 2 presents the unadjusted mean fitness z-scores by sample characteristics and weight status. To aid in interpretation, beta estimates reflect the standard deviations above or below overall age-sex normalized average. For instance, among male students, the average composite fitness score in the class III obesity group was approximately 1.1 standard deviations lower than the overall age-sex normalized average. Youth with healthy weight had the highest fitness z-scores compared to all other weight categories. Across all demographic categories, fitness z-scores decreased with increasing severity of obesity, with the lowest fitness scores observed in youth with class 3 obesity.

**Table 2:** Mean (SD) fitness z-scores scores by sample characteristics and weight status for NYC public school children 2010–2018 ^a^

Characteristic	Overall	Underweight	Healthy Weight	Overweight	Class I Obesity	Class II Obesity	Class III Obesity
Sex							
Male	0.06 (1.0)	0.25 (0.9)	0.33 (0.9)	0.02 (0.9)	−0.34 (0.9)	− 0.72 (0.9)	− 1.10 (0.9)
Female	0.08 (1.0)	0.32 (1.0)	0.29 (1.0)	−0.02 (0.9)	− 0.32 (0.9)	− 0.64 (0.9)	−0.95 (0.9)
Grades							
4th – 8th	0.06 (1.0)	0.33 (1.0)	0.32 (1.0)	−0.01 (0.9)	−0.34 (0.9)	− 0.69 (0.9)	− 1.04 (0.9)
9th – 12th	0.09 (1.0)	0.21 (0.9)	0.30 (0.9)	0.02 (0.9)	−0.32 (0.9)	−0.68 (0.9)	− 1.03 (0.9)
Race/ethnicity							
Non-Hispanic White	0.35 (1.0)	0.56 (1.0)	0.58 (1.0)	0.18 (0.9)	−0.21 (0.9)	−0.62 (0.9)	−1.00 (0.9)
Non-Hispanic Black	0.00 (1.0)	0.15 (0.9)	0.22 (0.9)	−0.01 (0.9)	−0.34 (0.9)	− 0.68 (0.9)	− 1.02 (0.9)
Hispanic	− 0.04 (1.0)	0.18 (0.9)	0.21 (0.9)	−0.05 (0.9)	− 0.37 (0.9)	− 0.71 (0.9)	− 1.06 (0.9)
Asian/Pacific Islander	0.17 (0.9)	0.29 (0.9)	0.34 (0.9)	0.00 (0.9)	−0.31 (0.8)	−0.64 (0.8)	− 0.92 (0.9)
Other ^b^	0.17 (1.0)	0.36 (1.0)	0.41 (1.0)	0.02 (1.0)	−0.33 (0.9)	−0.67 (0.9)	−1.00 (0.9)
Free/reduced meal status							
Yes	0.00 (1.0)	0.20 (0.9)	0.23 (0.9)	−0.04 (0.9)	−0.36 (0.9)	− 0.71 (0.9)	−1.05 (0.9)
No	0.26 (1.0)	0.47 (1.0)	0.49 (1.0)	0.12 (0.9)	−0.25 (0.9)	−0.62 (1.0)	− 0.99 (0.9)
Birth country							
Foreign born	0.07 (1.0)	0.31 (1.0)	0.32 (1.0)	0.00 (0.9)	−0.34 (0.9)	−0.69 (0.9)	−1.04 (0.9)
US born	0.06 (0.9)	0.20 (0.9)	0.26 (0.9)	−0.01 (0.9)	−0.33 (0.9)	− 0.67 (0.9)	−0.96 (0.9)
English language learner							
Yes	−0.21 (0.9)	−0.04 (0.9)	0.00 (0.9)	−0.27 (0.9)	− 0.56 (0.9)	−0.88 (0.8)	−1.21 (0.9)
No	0.10 (1.0)	0.31 (0.9)	0.33 (0.9)	0.02 (0.9)	−0.32 (0.9)	−0.67 (0.9)	−1.03 (0.9)

*Abbreviations*: *SD* Standard deviation, *NYC* New York City

^a^Increasing z-scores correspond with higher fitness

^b^Other includes mixed race, Native American, and refusal to provide

Table 3 shows the adjusted overall association between youth weight status and fitness as well as results from stratified analyses. In the overall model, compared to youth with healthy weight, increasing level of obesity was significantly associated with decreased fitness z-score: overweight (β = − 0.28, 95% CI: − 0.29 to − 0.28), class I obesity (β = − 0.60, 95% CI: − 0.60 to − 0.60), class II obesity (β = − 0.94, 95% CI: − 0.94 to − 0.93), and class III obesity (β = − 1.28; 95% CI: − 1.28 to − 1.27). In stratified analyses, the magnitude of this relationship was higher for boys and non-Hispanic White youth across all categories of overweight and obesity. Few differences were observed in the stratified models for grade level, country of birth, eligibility for free and reduced price meals, and English language learner status.

**Table 3:** Overall association between youth weight status and fitness and stratified by sample characteristics ^a^

	Underweight		Healthy weight		Overweight		Class I obesity		Class II obesity		Class III obesity	
	β	95% CI	β	95% CI	β	95% CI	β	95% CI	β	95% CI	β	95% CI
**Overall**	−0.04	− 0.05 to − 0.04	Reference		−0.28	− 0.29 to − 0.28	−0.60	− 0.60 to − 0.60	−0.94	− 0.94 to − 0.93	− 1.28	− 1.28 to − 1.27
**Sex**												
Boys	−0.09	− 0.10 to − 0.08	Reference		−0.30	− 0.30 to − 0.29	−0.65	− 0.65 to − 0.64	− 1.01	−1.01 to − 1.00	−1.38	− 1.39 to − 1.37
Girls	−0.01	0.00 to 0.01	Reference		−0.27	−0.27 to − 0.26	−0.54	− 0.55 to − 0.54	−0.85	− 0.85 to − 0.84	−1.15	− 1.16 to − 1.14
**Race/ethnicity**												
Non-Hispanic White	−0.04	− 0.06 to − 0.03	Reference		−0.36	− 0.36 to − 0.35	−0.72	− 0.73 to − 0.71	−1.10	− 1.11 to − 1.09	−1.45	− 1.48 to − 1.43
Non-Hispanic Black	−0.08	− 0.09 to − 0.07	Reference		−0.23	− 0.24 to − 0.23	−0.55	− 0.56 to − 0.55	−0.89	− 0.90 to − 0.88	−1.23	− 1.24 to − 1.22
Hispanic	−0.04	− 0.05 to − 0.03	Reference		−0.27	− 0.27 to − 0.26	−0.58	− 0.59 to − 0.58	−0.92	− 0.92 to − 0.91	−1.26	− 1.27 to − 1.25
Asian	−0.03	− 0.04 to − 0.02	Reference		−0.30	− 0.31 to − 0.29	−0.59	− 0.60 to − 0.59	−0.91	− 0.93 to − 0.90	−1.19	− 1.22 to − 1.15
Other	0.01	−0.03 to 0.05	Reference		−0.32	− 0.34 to − 0.29	−0.64	− 0.67 to − 0.61	−0.95	−1.00 to − 0.90	−1.35	− 1.43 to − 1.26
**Grade level**												
4th–8th	− 0.01	−0.02 to 0.00	Reference		−0.30	− 0.30 to − 0.30	−0.61	− 0.61 to − 0.61	−0.94	− 0.95 to − 0.94	−1.28	− 1.29 to − 1.27
9th–12th	−0.11	− 0.12 to − 0.10	Reference		−0.25	− 0.26 to − 0.25	−0.58	− 0.59 to − 0.58	−0.93	− 0.93 to − 0.92	−1.26	− 1.27 to − 1.25
**Birth country**												
Foreign born	− 0.04	−0.05 to − 0.03	Reference		−0.29	− 0.29 to − 0.28	−0.60	− 0.61 to − 0.60	−0.94	− 0.94 to − 0.93	− 1.28	− 1.29 to − 1.27
US born	−0.06	− 0.07 to − 0.05	Reference		−0.26	− 0.27 to − 0.25	−0.57	− 0.58 to − 0.56	−0.90	− 0.92 to − 0.89	−1.20	− 1.22 to − 1.17
**Free/reduced lunch**												
Eligible	−0.05	− 0.05 to − 0.04	Reference		−0.27	− 0.27 to − 0.27	−0.58	− 0.58 to − 0.58	−0.92	− 0.92 to − 0.91	−1.25	− 1.26 to − 1.24
Not Eligible	−0.05	− 0.05 to − 0.04	Reference		−0.31	− 0.32 to − 0.31	−0.65	− 0.66 to − 0.65	−0.99	−1.00 to − 0.98	−1.34	− 1.36 to − 1.33
**English learner**												
Yes	−0.04	− 0.06 to − 0.02	Reference		−0.26	− 0.27 to − 0.25	−0.54	− 0.55 to − 0.53	−0.86	− 0.87 to − 0.84	−1.18	− 1.21 to − 1.15
No	−0.04	− 0.05 to − 0.04	Reference		−0.28	− 0.29 to − 0.28	−0.60	− 0.61 to − 0.60	−0.94	− 0.95 to − 0.94	− 1.28	− 1.29 to − 1.27

*Abbreviations*: *CI* Confidence interval, *US* United States

^a^All models included BMI category, sex, grade, race/ethnicity, free/reduce price meal status, country of birth, English language learn status, and time unless stratified

## Discussion

The prevalence of youth with obesity is increasing, but little is known about the relationship between obesity and fitness in youth as weight status increases. Understanding this relationship across demographic subgroups can provide important targets for health promotion in youth with obesity and severe obesity, populations at high risk for chronic disease [18]. In this study of nearly one million NYC public school youth, we found a clear inverse dose response relationship between weight status and fitness, such that increasing levels of obesity corresponded to greater declines in fitness. Furthermore, the magnitude of this relationship was strongest for male and non-Hispanic White youth.

Findings from this study revealed an inverse dose response relationship between youth weight status and fitness, with the lowest fitness scores observed in youth with the most severe obesity. Youth with healthy weight had the highest fitness scores, followed by youth with underweight. The structural and functional limitations of obesity are likely one reason for the lower levels of fitness in these children and adolescents [29], as functional movement quality tends to be lower in youth with obesity [30]. For instance, in a study of 10–11 year old British children, children with overweight and obesity had significantly lower functional movement scores compared to children with healthy weight [31]. Lower functional movement can restrict daily PA, make certain movements and activities difficult or painful, and overall reduce youth’s weight-related quality of life, especially in youth with severe obesity. Another contributing factor may be that weight based stigma and internalized bias contribute to avoidance of PA in youth with obesity. Children and adolescents who experience teasing and bullying related to weight avoid PA, participate less in physical education class, and are less likely to participate in organized sports [32]. These factors likely results in limited engagement in fitness promoting physical activities, contributing to lower overall fitness in youth with obesity.

This study also found that that weight status-fitness relationship was modified by sex, such that with increasing weight status boys had lower fitness compared to girls. This finding is contrary to what would be expected based on national PA data where boys tend to be more active than girls [2]. However, temporal trends in fitness indicate that worldwide, boys have experienced greater declines in fitness over time, although this does not account for obesity [6]. Furthermore, a cross sectional study with Australian children found that the inverse relationship between weight status and fitness was stronger for boys, such that increasing obesity corresponded with greater declines in cardiorespiratory fitness in boys compared to girls [33]. One possible explanation for this relationship may be sex differences in motivation and effort in completing the Fitnessgram, or that boys may experience greater stigma around fitness and PA compared to girls [34]. Future research will need to continue to explore the association between weight status and fitness in youth to examine nuances in this relationship across sex and weight categories.

While non-Hispanic White youth had the highest fitness scores of any other racial or ethnic group, the magnitude of the weight status-fitness relationship was strongest in this group, which was surprising given well-documented disparities in obesity and PA in non-Hispanic Black and Hispanic populations [9, 19]. It is likely that this may be attributed to the difference in fitness scores among non-Hispanic White youth with the most severe obesity and healthy weight relative to other racial/ethnic subgroups. Thus, the magnitude of the weight status-fitness association is largest in the non-Hispanic White group because the average fitness level in the reference group (youth with healthy weight) relative to youth with severe obesity in non-Hispanic White youth is highest. Another possible explanation may be that minority youth participate in more active transportation (e.g., walking and biking) and use public transportation more frequently, which could contribute to increased fitness [35]. In a study using data from the National Health and Nutrition Examination Survey (NHANES), non-Hispanic Black and Hispanic youth were more likely to engage in active transport compared to non-Hispanic White youth [36]. Additionally, findings from another US-based study show that minority youth are more likely to use public transportation, which was associated with increased moderate to vigorous PA [37]. It is also possible that to further understand racial/ethnic difference in fitness, we may need to consider interaction with other demographic variables (e.g., race/ethnicity and gender).

Interestingly, when examining the mean fitness z-scores, English language learner students had the lowest fitness z-scores of any demographic subgroup; however, after adjustment for other sample characteristics, the magnitude of this relationship was smaller compared to youth who were not English language learner students. Studies of immigrant children show heterogeneity in physical activity, health status, and obesity risk among different immigrant groups [38–41]. It may be that some of the social cohesion and cultural practices within ethnic enclaves can be beneficial or detrimental to health promotion in children [42]. Future research should continue to explore cultural and neighborhood factors associated with immigration status that may explain the weight status-fitness relationship.

The findings in this study highlight the importance of promoting fitness in youth particularly with high weight status. For youth with obesity, behavioral lifestyle interventions generally results in modest decreases in body weight and much of the weight lost is often regained following the conclusion of treatment [43]. However, even in the absence of weight loss, improvements in fitness can have positive effects on current and future health [44, 45]. Providers and public health professionals should work with children and adolescents to set realistic, safe, and sustainable exercise goals and provide access to resources such as exercise-focused afterschool programs or community-based program run by organizations like Parks and Recreation [46, 47]. For instance, a study of an afterschool fitness program showed improvements in fitness and percent body fat [48], while a study of a Parks and Recreation fitness program showed increases in fitness and reductions in cardiovascular disease risk for children with severe obesity [49]. Advocating for schools to institute fitness related programs and policies is another approach to promoting fitness. For instance, in NYC, the DOE’s Office of School Wellness offers a number of fitness related programs designed to incorporate fitness breaks throughout the day, provide after school support, and peer mentorship [50–52].

This study had a number of strengths including the use of longitudinal data with a lagged fitness outcome, a large, diverse sample representing populations at greater risk for health disparities, and adequate representation of youth with severe obesity; however, it is was not without limitations. Because the NYC Fitnessgram is assessed only in public school youth, findings may not generalize to youth in private, charter, or special educations schools. Additionally, findings may not be generalizable to youth who do not live in large, urban settings. Another limitation is that we did not use the VO2 peak testing to assess fitness, which is considered the gold standard for assessing fitness and is able to assess fitness independent of body weight. Furthermore, although the Fitnessgram is a valid and reliable assessment of child fitness, NYC Fitnessgram testing was not conducted in a research environment, so it is possible there are differences in the delivery of the testing. To help ensure fidelity, NYC Fitnessgram administrators are provide with training and educational materials. Additionally, while BMI is often used as population measure of adiposity, it is not without limitations. Some individuals may have been misclassified, particularly among the overweight category, based on racial and ethnic differences in body composition as well as differences in lean body mass (i.e., athletes). However, given the large sample, it is likely that this potential misclassification did not significantly affect our findings. Finally, we were unable to take into account social and neighborhood factors that may contribute to differences in youth fitness. Future studies should seek to better understand these factors within youth with severe obesity.

## Conclusion

Our study found that among almost one million NYC public school youth followed for 8 years, higher weight status was associated with poorer fitness. This relationship was strongest in magnitude for male and non-Hispanic White students. These data highlight opportunities for targeted clinical and public health interventions to improve both the weight and fitness of children and adolescents with obesity in order to reduce their risk for future chronic disease.

## Data Availability

The datasets generated and/or analyzed during the current study are not publicly available as it contains identifiable information but are available from the corresponding author on reasonable request.

## Abbreviations

BMI: Body Mass Index
CDC: Centers for Disease Control and Prevention
DOE: Department of Education
DOHMH: Department of Health and Mental Hygiene
NHANES: National Health and Nutrition Examination Survey
NYC: New York City
PA: Physical activity
PACER: Progressive Aerobic Cardiovascular Endurance Run
US: United States
